# A temporal and spatial contribution of asparaginase to asparagine catabolism during development of rice grains

**DOI:** 10.1186/s12284-017-0143-8

**Published:** 2017-01-25

**Authors:** Yui Yabuki, Miwa Ohashi, Fumi Imagawa, Keiki Ishiyama, Marcel Pascal Beier, Noriyuki Konishi, Toshiko Umetsu-Ohashi, Toshihiko Hayakawa, Tomoyuki Yamaya, Soichi Kojima

**Affiliations:** Graduate School of Agricultural Science, Tohoku University, 468-1 Aoba, Aramaki, Sendai, 9800845 Japan

**Keywords:** Asparagine, Rice, Asparaginase, Nitrogen, Translocation

## Abstract

**Background:**

Asparagine is one of the most dominant organic nitrogen compounds in phloem and xylem sap in a wide range of plant species. Asparaginase (ASNase; EC, 3.5.1.1) catabolizes asparagine into aspartate and ammonium; therefore, it is suggested to play a key role in asparagine metabolism within legume sink organs. However, the metabolic fate of asparagine in source and sink organs during rice seed production remains to be elucidated. Therefore, the main objective of this study is to investigate the asparagine metabolism in a temporal and spatial manner during rice seed production.

**Results:**

For this purpose, the expression of genes involved in asparagine catabolism, such as asparaginase1 (*OsASNase1*) and 2 (*OsASNase2*), were quantitatively measured, and contents of asparagine, aspartate and ammonium ions were determined in sink and source organs during spikelet ripening. Quantitative real-time PCR and *in situ* localization studies determined that *OsASNase2* is expressed in the dorsal vascular bundles and nucellar projection of developing grains, as well as in mesophyll and phloem companion cells of senescent flag leaves. Amino acid measurements revealed that the aspartate concentration is higher than asparagine in both source and sink organs.

**Conclusion:**

This work suggests that asparaginase dependent asparagine catabolism occurred not only in sink but also in source organs.

**Electronic supplementary material:**

The online version of this article (doi:10.1186/s12284-017-0143-8) contains supplementary material, which is available to authorized users.

## Background

Food production is dependent on nitrogen translocation through the phloem in plants, since most grain nitrogen is remobilized from source organs during senescence in crops (Szpak 2014). The ratio of the source organ nitrogen to the remobilized grain nitrogen is suggested to be 85% in maize (Ta and Weiland 1992), 100% in wheat (Martre et al. 2003; Tahir and Nakata 2005), and 65% in rice (Mae and Ohira 1981). Plant phloem transports nitrogen compounds from source to sink organs (Raven and Smith 1976).

Asn is one of the most dominant organic nitrogen compounds for translocation, as well as Gln, in many plant species (Lea and Ireland 1999; Lea et al. 2007). Asparaginase (ASNase; EC, 3.5.1.1) catabolizes Asn into Asp and NH_4_^+^ (Lea et al. 2007), while asparagine synthetase (AS; EC, 6.3.5.4) generates Asn (Canovas et al. 2007; Lea et al. 2007). Genes encoding ASNase, has been cloned from several plant species like *Lupinus arboreus* (Lough et al. 1992), *Lupinus angustifolius* (Dickson et al. 1992)*, Arabidopsis, thaliana* (Hejazi et al. 2002)*, Lupinus luteus* (Borek et al. 2004)*,* scot pine (Canas et al. 2007), soybean (Cho et al. 2007), and more recently *Lotus japonicas* (Credali et al. 2011).

In higher plants, phylogenetic analysis of the amino acid sequence indicated that two types of ASNase were categorized into two evolutionally distinct subfamilies; a K^+^-dependent and an independent type (Bruneau et al. 2006). The expression of two *ASNase* genes was temporally and spatially regulated. For example, quantitative real-time polymerase chain reaction (qPCR) was able to provide, not only the overlapping expression of two *ASNase* genes in various tissues of Arabidopsis during its growth, but also the quantitative ratio between K^+^-dependent and K^+^-independent ASNase (Bruneau et al. 2006). Transcript levels of Arabidopsis K^+^-independent ASNase (At5g08100, ASPGA1) were much higher than that of K^+^-dependent ASNase (At3g16150, ASPGB1) in flowers, siliques, seedlings and rosette leaves (Bruneau et al. 2006). Although the ratio of ASNase isozymes is not clear in other plant species except Arabidopsis, the previous articles support the key role of ASNase in sink tissues, especially in legume developing seeds and fruits (Atkins et al. 1975; Bruneau et al. 2006; Murray and Kennedy 1980; Sieciechowicz et al. 1988).

However, there is a clear difference between legumes and gramineaes in regard to nitrogen fixation. It may reflect the difference of phloem sap composition in two plant groups. For example, Asn accounts for approximately 40% or more to the total free amino acid concentration in the phloem sap collected from the petiole of *Lupinus albus* (Pate et al. 1979), while approximately only to 12% of the free amino acid concentration in the phloem sap collected from the uppermost internode of rice plants (Hayashi and Chino 1990). Therefore, it is worth to study Asn catabolism in gramineae. This work determined the temporal and spatial distribution of transcripts for the two genes related to Asn catabolism, *OsASNase* in rice plants during the development of spikelets.

## Results

### The concentration of Asp is higher than that of Asn in both source and sink organs

**Fig. 1:**
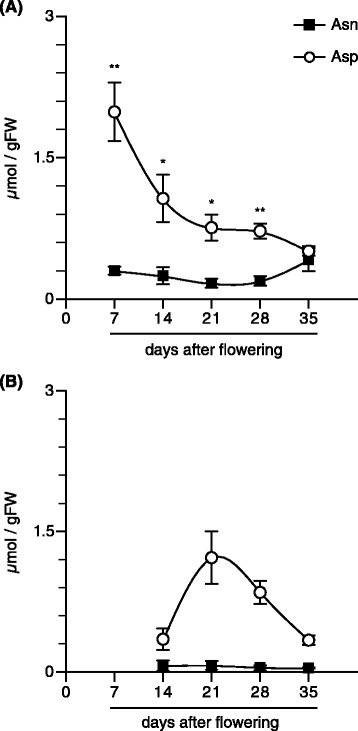
The contents of free aspartate and asparagine in spikelets and flag leaves in rice. The apical spikelets (**a**) on the primary branches at the positions from the first (top) to the fifth, and flag leaves (**b**) were harvested during the ripening period from 7 to 35 and 14 to 35 days after flowering, respectively. Means of three to four independent samples and standard error values (*n* = 3 to 4) are indicated. Significant differences between aspartate and asparagine identified by Student’s *t*-test are marked with asterisks: *P < 0.05, **P < 0.01

Firstly, the concentrations of Asn and Asp were quantified every seven days in developing spikelet (Fig. 1a) and flag leaf (Fig. 1b) at 7 to 35 and 14 to 35 days after flowering (DAF), respectively (Fig. 1).

In developing spikelets, the concentration of Asp was six-times higher than that of Asn at 7 DAF, and then decreased linearly during the development, while Asn concentration did not change dramatically (Fig. 1a). Asn and Asp concentration became almost equal at 35 DAF.

The Asp concentration in flag leaves was always higher than that of Asn at all harvest stages with a peak at 21 DAF (Fig. 1b). Asp had a ten-times higher concentration than Asn at 21 DAF.

### Rice expresses both potassium dependent and independent *OsASNase* genes

**Fig. 2:**
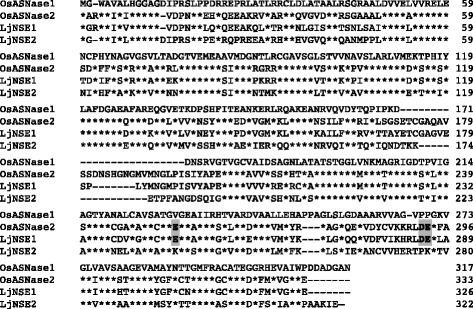
Alignment of the deduced amino acid sequences between ASNase1, ASNase2, LjNSE1 and LjNSE2 peptides. The deduced amino acid sequences are represented in the one-letter code for each amino acid. Asterisk symbols (*) in ASNase2, NSE1 and NSE2 lines indicate identical amino acids of ASNase1. Proposed putative K^+^ binding-sites are indicated with bold letters on gray columns, respectively. *OsASNase1* is identical to Os03g0597600, while *OsASNase2* is Os04g0650700, respectively

Two *ASNase* genes*, OsASNase1* and *OsASNase2*, were identified in the RAP-DB. No other homologous genes were found. The deduced amino acid sequences coded two putative *OsASNase* genes, which were compared to two *Lotus ASNase* genes. The Lotus *ASNase* genes are known to encode for functional enzymes via recombinant protein expression analyses (Credali et al. 2011). The alignment of deduced amino acid sequences for ASNase1, ASNase2, LjNSE1 and LjNSE2 was shown in Fig. 2. The alignment indicated a potential K^+^-binding domain conserved only in ASNase2 which leads to the suggestion that ASNase1 is a K^+^-independent ASNase, whereas ASNase2 could be a K^+^-dependent one. The alignment of deduced amino acid sequences showed high homology between *OsASNase* genes and their corresponding *Lotus ASNase* genes, *LjNSE1* and *2*. ASNase1 showed 73% identity and 90% similarity to LjNSE2 on a basis of amino acid sequences, while ASNase2 showed 77% identity and 92% similarity to LjNSE1.

### *OsASNase2* localization overlapped with *OsGS1* in vascular tissues of developing rice spikelets

**Fig. 3:**
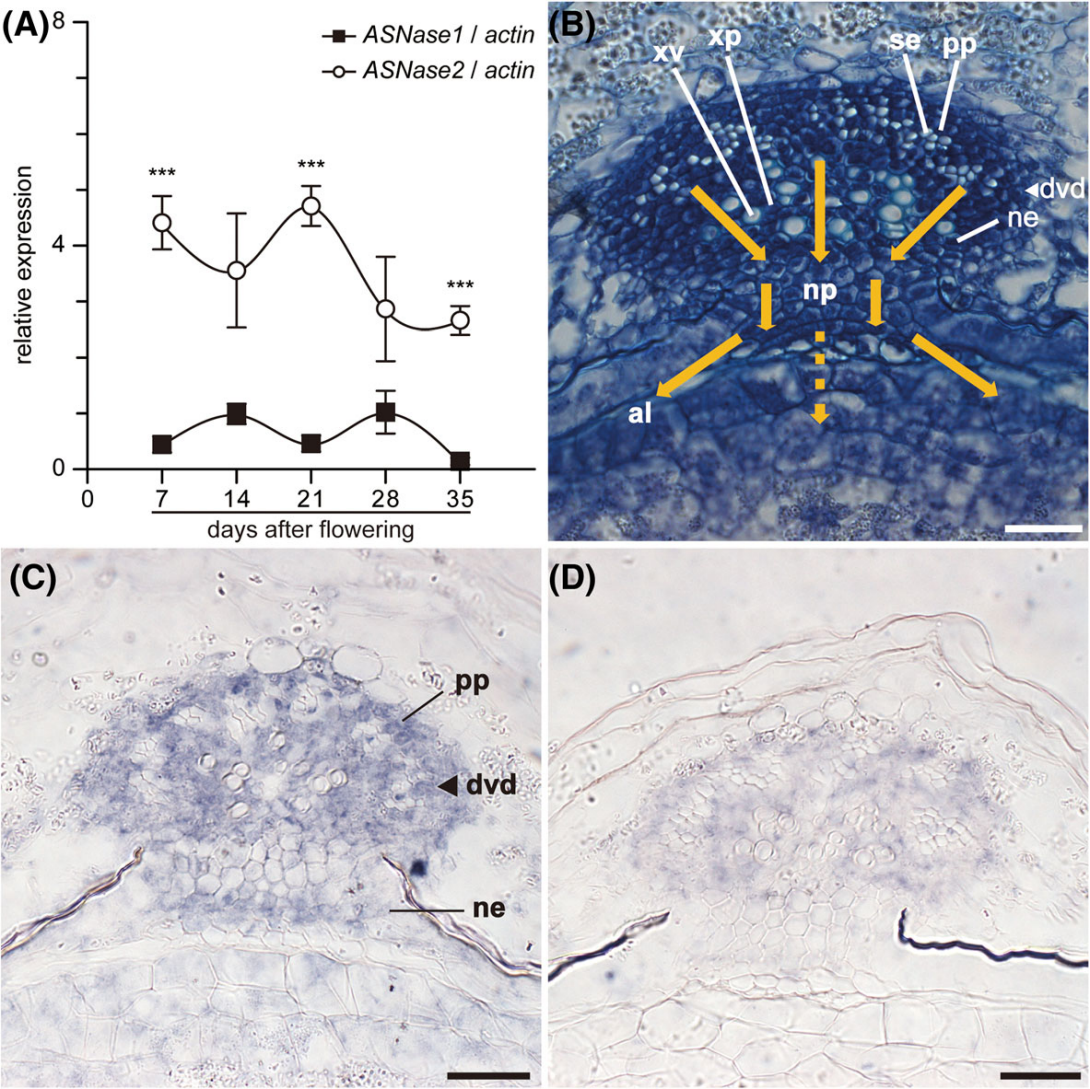
Quantitative real-time PCR analysis and *in situ* hybridization for *OsASNase1* and *OsASNase2* in rice spikelets. The apical spikelets on the primary branches at the positions from the first (top) to the fifth were harvested during the ripening period from 7 to 35 days after flowering, respectively. The detection of transcript for *OsASNase1* (filled square) and *OsASNase2* (opened circle) was conducted in spikelets using quantitative real-time PCR (**a**). Quantitative real-time PCR analyses were performed using gene-specific primers for *OsASNase1, OsASNase2* and *actin*, like described in Table 1. The relative contents of these transcripts were normalized against *actin* transcript. Means of four independent samples and standard error values (*n* = 4) are indicated. Significant differences between *OsASNase1* and *OsASNase2* identified by Student’s *t*-test are marked with asterisks: ***P < 0.001. *In situ* detection of *OsASNase2* transcript was conducted in spikelets at 14 days after flowering (**c** and **d**). The antisense probe for *OsASNase2* transcript was hybridized with the sections from spikelets (**c**). The sense probe for *OsASNase2* transcript was hybridized with the sections as negative control (**d**). The staining reaction was performed for 16 h. The Structure of tissues was visualized with toluidine blue staining (**b**). Abbreviations: al, aleurone layer; dvb, dorsal vascular bundle; ne, nucellar epidermis; np, nucellar projection; pp, phloem parenchyma cell; se, sieve element; xp, xylem parenchyma cell; and xv, xylem vessel element. Bars for (**b**) to (**d**) are 30 μm, respectively

The expression of two *OsASNase* genes was characterized in developing rice spikelets. Rice spikelets were harvested at every 7 DAF. The qPCR analysis indicated that the expression of *OsASNase2* was much higher than that of *OsASNase1* at all harvest points, but the two *OsASNase* genes were constantly expressed in rice spikelets during the maturation (Fig. 3a).

*OsASNase2* transcript accumulation was detected in the dorsal vascular bundle, especially in phloem parenchyma cells and the nucellar projection in spikelets, using antisense probes for *in situ* hybridization analysis (Fig. 3c). When the *OsASNase2* sense probe was used, only weak signals as background level were observed (Fig. 3d). Toluidine blue O staining visualized the structure of a developing rice spikelet, and the arrows are representing the metabolite flow from phloem sap (Fig. 3b) (Matsuda et al. 1979). Neither sense nor antisense probes for *OsASNase1* transcripts provided a clear staining (result not shown).

**Fig. 4:**
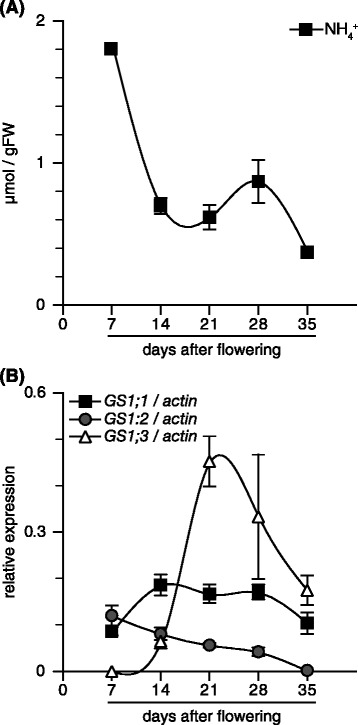
The content of NH_4_^+^ and transcriptional levels of *OsGS1;1*, *OsGS1;2* and *OsGS1;3* in rice spikelets. The apical spikelets on the primary branches at the positions from the first (top) to the fifth were harvested during the ripening period from 7 to 35 days after flowering. NH4+ concentration was measured (**a**). The detection of transcript for *OsGS1;1* (filled square), *OsGS1;2* (opened circle) and *OSGS1;3* (opened triangle) was conducted in spikelets using quantitative real-time PCR (**b**). Quantitative real-time PCR analyses were performed using gene-specific primers for *OsGS1;1*, *OsGS1;2*, *OsGS1;3* and *actin*, respectively, as described in Table 1. The relative contents of these transcripts were normalized against *actin* transcript. Means of four independent samples and standard error values (*n* = 4) are indicated

Constitutive *OsASNase* expression suggested the accumulation of ammonium; therefore, free ammonium concentration was measured in developing spikelets (Fig. 4a). Indeed, the free ammonium concentration in spikelets showed a peak at 7 DAF. During the spikelet maturation, the free ammonium concentration decreased. At 35 DAF, the free ammonium concentration was reduced by approximately 80% in comparison to the peak value.

GS1 seemed to assimilate free ammonium in developing spikelets, since chloroplastic GS (GS2) showed no high expression in the organ (Yamaya and Kusano 2014). Three cytosolic *OsGS1* genes were expressed in spikelets during the seed maturation (Fig. 4b). The *OsGS1;1* expression was relatively constitutive, while *OsGS1;2* expression showed a linear decrease during the maturation (Fig. 4b). *OsGS1;3* transcripts were rarely detectable at the beginning of anthesis, however, the amount sharply increased until 21 DAF, and it afterwards declined (Fig. 4b).

### *OsASNase2* is highly expressed in mesophyll and phloem companion cells of senescing flag leaves

**Fig. 5:**
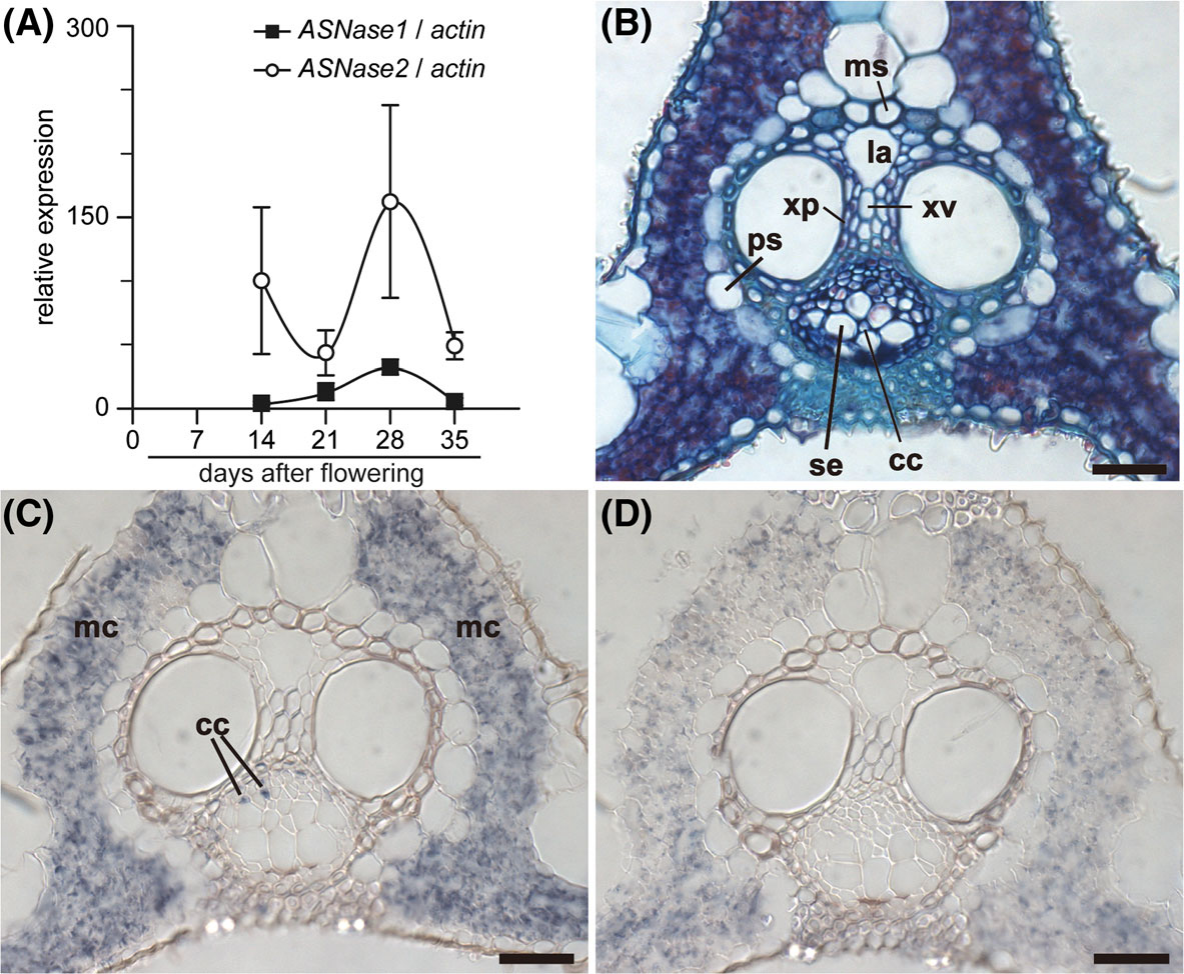
Quantitative real-time PCR analysis and *in situ* hybridization for *OsASNase1* and *OsASNase2* in flag leaves. The flag leaves were harvested during the ripening period from 14 to 35 days after flowering. The detection of transcript for *OsASNase1* (filled square) and *OsASNase2* (opened circle) were conducted in flag leaves using quantitative real-time PCR (**a**). Quantitative real-time PCR was performed using gene-specific primers for *OsASNase1, OsASNase2* and *actin,* like described in Table 1. The relative contents of these transcripts were normalized against *actin* transcript. Means of four independent samples and standard error values (*n* = 3) are indicated. *In situ* detection of *OsASNase2* transcript was conducted in flag leaves at 14 days after flowering (**c** and **d**). The antisense probe for *OsASNase2* transcript was hybridized with the sections from flag leaves (**c**). The sense probe for *OsASNase2* transcript was hybridized with the sections as negative control (**d**). The staining reaction was performed for 16 h. The structure of tissues was visualized with toluidine blue staining (**b**). Abbreviations: cc, companion cell; la, protoxylem lacuna; ms, mestome sheath cell; mc, mesophyll cell; ps, parenchyma sheath; se, sieve element and xv, xylem vessel element. Bars for (**b**) to (**d**) are 30 μm, respectively. Significant differences between *OsASNase1* and *OsASNase2* identified by Student’s *t*-test are marked with asterisks: *P < 0.05

The expression of two *OsASNase* genes was compared in sink and source organs. Senescent flag leaves were harvested at every 7 DAF. The qPCR showed that the expression of *OsASNase2* was higher than that of *OsASNase1* in flag leaves at all harvested stages (Fig. 5a).

The *OsASNase2* transcript dependent signals were detected in mesophyll cells and phloem companion cells of flag leaves at 14 DAF (Fig. 5c). Only a faint staining was observed when sense strand probes were used (Fig. 5d), while *OsASNase1* antisense probes did not show obvious staining by *in situ* hybridization (data not shown).

## Discussion

### Spatial and temporal distribution of asparaginase2 suggests its contribution to asparagine sink-source translocation in rice plant

As Asp is the common precursor of the essential amino acids lysine, threonine, methionine and isoleucine in higher plants (Azevedo et al. 2006), Asn has to be catabolized into Asp and ammonium in both flag leaves and spikelets. The previous work (Hayashi and Chino 1990) suggested the concentration of Asp is five-times higher than that of Ass in wheat phloem sap. Conversely, this work showed the dynamic changes of Asp concentration in both sink and source organs during seed development. Asn concentration was relatively stable. Furthermore, spikelets contained a certain amount of free ammonium (Fig. 4a). Since qPCR analyses indicated that *OsASNase2* was the major ASNase isoform in shoots (Additional file 1: Figure S1), which is especially highly expressed in spikelets and flag leaves, we hypothesized that ASNase2 is involved in Asn catabolism in those organs.

**Fig. 6:**
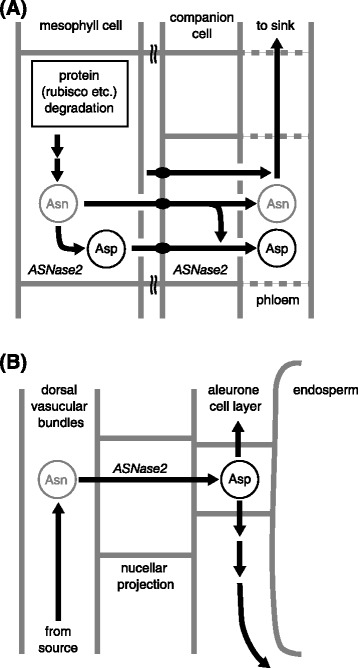
Schematic model for the degradation of asparagine by ASNase2 in rice flag leaves and spikelets. ASNase2 has a role in the reutilization of asparagine in source (flag leaves) and sink (spikelets) organs. Box-shaped panel indicates the proteins in mesophyll cell (**a**). Round-shaped panels indicate the pools for free amino acids (**a** and **b**). Abbreviations: Asn, asparagine; Asp, aspartate; Gln, glutamine and Glu, glutamate

The function of ASNase in sink and source organs could be hypothesized for rice plants (Fig. 6). Proteins, which are mainly related to photosynthesis, are degraded and release huge amounts of Asn in source organs, for example in senescent leaves (Fig. 6a) (Mae et al. 1985). ASNase2 catabolize some moiety of Asn molecule into Asp and ammonium in mesophyll cells and phloem companion cells. In sink organ, like developing spikelets (Fig. 6b), ASNase2 catabolizes Asn that is transported in dorsal vascular bundles. This catabolism takes place in phloem parenchyma cells of dorsal vascular bundles and nucellar projections. Asp is mostly moved along aleurone cell layers through the nucellar projection, but partially transported into endosperm for the preparation of seed storage proteins (Hoshikawa 1989). Our localization study in this work expands previous works, since the localization of ASNase was only investigated in Scots pine (Canas et al. 2007) or Arabidopsis (Ivanov et al. 2012), but not in rice. ASNase localization in rice suggested that ASNase could relate to the nitrogen remobilization: firstly, ASNase was localized in developing rice spikelets, secondly, it localized in phloem companion cells in senescing leaves, thirdly, it also localized in mesophyll cells. Pine ASNase was localized in cambial cells of hypocotyls (Canas et al. 2007). Arabidopsis *ASNase* promoter dependent GUS activity was found in leaf vascular tissues, inflorescence stems and developing seeds in soil grown plants (Ivanov et al. 2012). The variety of ASNase localization may reflect the broad physiological function of this enzyme on wide plant species.

Previous articles pointed that Gln dependent AS is the main enzyme used for the synthesis of the Asn in higher plants (Canovas et al. 2007; Lea et al. 2007). Rice genome sequence analysis (Sequencing Project International Rice G 2005) revealed that the rice genome encodes two *OsAS* genes, *OsAS1* and *OsAS2*.

Grain ASNase2 seemed to catabolize Asn that was transported from source organs, since the expression of both *OsAS* genes (Additional file 2: Figure S2) were surprisingly much lower than those of two *OsASNase* genes (Fig. 3a and 5a) in all tested conditions. In fact, our previous research indicated that AS protein was rarely detectable in rice leaf blades (Nakano et al. 2000).

Conversely, foliar ASNase2 seemed to catabolize Asn originated from protein degradation in source organs. Proteins should be a major source for nitrogen translocation, since their nitrogen account for approximately 70% of the total nitrogen content in senescent leaves (Mae et al. 1985). Especially, Ribulose-1,5-bisphosphate carboxylase/oxygenase (Rubisco) was considered as an important nitrogen source for translocation, since this protein accounted for about half of the soluble proteins in the mature rice leaves (Mae et al. 1985).

Plant Rubisco consists of large and small subunits (Suzuki et al. 2007). Five Rubisco small subunit genes encode identical amino acid sequences except their transit peptide (Suzuki et al. 2007). Indeed, five and fifteen Asn residues were found in small and large subunits, respectively, those vast amounts of Asn molecules could be a substrate for ASNase2 during leaf senescence. Rice leaves contain approximately 20 mg soluble protein/g fresh weight (gFW) before developing spikelets (Kamachi et al. 1991). Since the half amount of soluble proteins is Rubisco (Mae et al. 1985), rice senescing leaves may contain approximately 2.8 μmole Asn/gFW.

Reverse genetic approaches could provide direct evidence for gene functions to support histochemical studies. However, rice ASNase defective mutants have not been isolated yet. Recently, Arabidopsis T-DNA insertion lines for ASNase1 and ASNase2 were isolated, respectively, and then, two insertion lines were crossed, and double insertion lines were generated (Ivanov et al. 2012). The characteristics of these double insertion lines could be summarized in two points. Firstly, double insertion lines accumulated free Asn. Secondary, a reduction of available nitrogen slightly enlarged the size of the mutant in comparison to the corresponding genetic background. Despite previous articles suggested the key role of ASNase in sink Asn catabolism, Arabidopsis ASNase mutants showed surprisingly no obvious phenotype in seed productivity. For a mild phenotype, low Asn concentration in *Brassica* phloem sap and alternative Asn catabolism pathways were suggested (Ivanov et al. 2012). The rice yield consists of three factors; those are, number of grains, ripening ratio and seed weight. Although seed size may reflect seed weight, the diversity of plant seed number is much larger than that of seed size (Sadras 2007). Therefore, the comparison of yield components between wild-type and ASNase defective mutant rice may provide the evidence for our suggested model in future.

### Grain GS1 could assimilate ammonium released from ASNase reaction

All three GS1 genes were expressed in developing spikelets (Fig. 4b), in addition, *OsGS1;1* transcripts localized in dorsal vascular bundles, nucellar projection and aleurone cell layers (Additional file 3: Figure S3). This result is consistent with the results in previous studies (Thiel et al. 2008). Microdissection-based transcriptome studies indicated the expression of several GS1 genes in nucellar projections of barley, one of the most important gramineae species (Thiel et al. 2008). Nucellar projections play an important role for endosperm growth and development (Matsuda et al. 1979; Martre et al. 2003). In this regard, free ammonium in nucellar projections should be quickly assimilated for an efficient nitrogen use. Therefore, GS1 seemed to be coupled with ASNase2 for the assimilation of ammonium derived from Asn degradation conducted by ASNase in developing rice grains. The functional association of both enzymes was consistent with the results from previous work; it showed the overlapping localization of both enzymes in hypocotyls of pine seedlings (Canas et al. 2007) for vascular development.

## Methods

### Plant materials

*Oryza sativa* L. cv. Nipponbare was used for all experiments. Seeds were germinated in distilled water at 30 °C for 48 h in the dark. Each germinated seed was planted on a synthetic culture soil (Type-L, Sanken-Soil Corporation, Iwate, Japan) in a small container. Twenty days later, single seedlings were transplanted into a 24 L plastic pot with 42 g of slow-release fertilizer (N,16%; P,16%; K,16%: Coop Chemical Co., Tokyo, Japan) and grown in a greenhouse with irrigation, as described previously (Obara et al. 2001). Flowering of the ear occurred between 117–143 days after sowing and the ears were tagged on that day. The apical spikelets on the primary branches at the positions from the first (top) to the fifth as well as the flag leaves were harvested during the ripening period from 7 to 42 and 14 to 42 days after flowering (DAF), respectively, as described by Hayakawa et al. (1993). For qPCR and *in situ* hybridization analyses to identify the cellular expression for *OsASNase1* (accession No. AK069458, Rice Annotation Project Database (RAP-DB, http://rapdb.dna.affrc.go.jp/) code Os03g0597600), *OsASNase2* (accession No. AK121820, RAP-DB code Os04g0650700), *OsGS1;1* (accession No. AK109397, RAP-DB code Os02g0735200)*, OsGS1;2* (accession No. X14244, RAP-DB code Os03g0223400)*, OsGS1;3* (accession No. AK099290, RAP-DB code Os03g0712800), *OsAS1* (accession No. CI197925, RAP-code Os03g0291500) and *OsAS2* (accession No. D83378, RAP-code Os06g0265000) genes, the spikelets and flag leaves were harvested at 14 DAF.

### Free amino acid measurement

Free amino acids and NH_4_^+^ contents in spikelet and flag leaf were measured according to our previous experiment with slightly modification (Kojima et al. 2014). Spikelets and flag leaves were harvested in 2.0 mL safe lock tube (Eppendorf Co., Ltd., Tokyo, Japan) with zirconia beads and stored at −80 °C until free amino acid extraction. The plant samples were pre-chilled in liquid nitrogen, and then milled with Tissue Lyser II (Qiagen, K. K., Tokyo, Japan) at 25 hertz for 2 min. Powdered samples were resolved in 10 mM HCl, mixed in Tissue Lyser II at 25 hertz for 1 min, and centrifuged at 20,500 *g* for 15 min at room temperature. The supernatant (ca. 100 μl) was transferred to the prepared Amicon Ultra-0.5 mL Centrifugal Filters (Millipore, Bedford, MA, USA) on 1.5 mL tubes, and centrifuged at 20,500 *g* for 15 min at room temperature. The flow-through was collected and stored at −30 °C until labeling. The AccQ•Tag Ultra Derivatization Kit (Nihon Waters K. K., Tokyo, Japan) was used for derivatization of free amino acids and ammonium. AccQ•Tag Ultra Borate Buffer (30 μl) and the Amino Acids Mixture Standard Solution, Type H (Wako Pure Chemical Industries, Ltd., Osaka, Japan) or extracted sample (10 μl) were vortexed briefly, and AccQ•Tag Reagent (10 μl) was added and vortexed again for 10 s. After a 1 min incubation at room temperature, samples were heated in a block incubator for 10 min at 55 °C. AccQ•Tag labeled samples were kept at room temperature and measured with ACQUITY UPLC H-Class (Nihon Waters K. K.).

### Cloning of rice *ASNase* cDNAs

Molecular biological experiments were carried out according to our previous protocols (Ishiyama et al. 2004; Ohashi et al. 2015). The coding sequences of cDNAs encoding *ASNase* were isolated by reverse transcriptase (RT) PCR for first strand DNA synthesis using specific primer pairs for *OsASNase1* and *OsASNase2*, respectively (Table 1), designed according to the nucleotide sequence of these genes in rice. Total RNA was extracted using the RNeasy plant kit (Qiagen, K. K). Reverse transcription was carried out using the PrimeScript® RT reagent Kit with gDNA Eraser (Takara Bio Inc. Otsu, Shiga, Japan), followed by a PCR with KOD Fx NEO DNA polymerase (Toyobo, Tokyo, Japan). The amplified PCR products were cloned into pCR-BluntII-TOPO (Life Technologies Corporation, Carlsbad, CA USA) and fully sequenced.

**Table 1:** List for gene specific primers used in this work

Name	Sequence
OsASNase1_CDS F	ATAGATAGATCCATGGGGTGGGCGGTCGCGCTGCA
OsASNase1_CDS R	GAAGATTGCTGAACGAAGATCAATTAGCACCATCA
OsASNase2_CDS F	GCCTAGGAAATACTACGTCGATGGCGAGGTGGGCC
OsASNase2_CDS R	CACGCACAAAGGCTGCCCACCTCACTCCCAGATGC
ASNase1_real F	GGTGCAGGTGGATTACACCCAACCA
ASNase1_real R	CCCGCCGGTGGACGTCGCCGTCGCG
ASNase2_real_F_01	CATCTTGTTTGACTACCGTATCCCG
ASNase2_real_R_01	GATGGGGAGGCCGTTCATCACCATG
OsActin_F	CTTCATAGGAATGGAAGCTGCGGGTA
OsActin_R	CGACCACCTTGATCTTCATGCTGCTA
OsASNase1_3′UTR F	GACGCTGATGGTGCTAATTGATCTTCGTTCAGCAA
OsASNase1_3′UTR R	CTTAATTGAATTTGGATATAAATTGAGCTGATTGT
OsASNase2_3′UTR F	GATTCATGGAGGTCGGCATCTGGGAGTGAGGTGGG
OsASNase2_3′UTR R	CCATCATCGTCATTATTCAAGCCCACCTCCAATCT

### Quantitative real-time PCR

Total RNA was extracted from liquid nitrogen frozen spikelets and flag leaf with the RNeasy plant mini kit (Qiagen, K. K.). Three to four independent experiments were carried out. The extraction was performed following manufacturer’s instructions. Reverse transcription and DNase-treatment were carried out using a PrimeScript® RT reagent Kit with gDNA Eraser (Takara Bio Inc.) with 500 ng of total RNA in a 20 μl final volume, according to manufacturer’s instructions. After 5 min heat inactivation of the enzyme, the quality of extracted RNA samples was quantified using the NanoDrop 1000 spectrophotometer (NanoDrop, LMS CO., LTD. Tokyo, Japan). The A260/280 ratio is generally between 1.9 and 2.0. Since a melting curve was performed as standard, a contamination would be visible as additional peak. Aliquots of the resulting RT reaction products were used as templates for PCR analysis. Prepared cDNA samples were stored in 1.5 mL tubes (Eppendorf) at −20 °C. The qPCR analysis was conducted using gene-specific primers (Table 1). Gene-specific primers were designed using the DNASIS software (Hitachi Solutions, Ltd., Tokyo, Japan). In *silico* screening was performed with NCBI Blast (http://blast.ncbi.nlm.nih.gov/Blast.cgi). Multiplex qPCR was not performed. Primers were purchased from Eurofins MWG Operon Inc., Tokyo, Japan. The PCR products were quantified using the Light Cycler® 480 (Roche Diagnostics K.K., Tokyo, Japan) according to the following program: 10 s at 95 °C, followed by 50 cycles of 95 °C for 5 s, and 60 °C for 34 s. The reaction mixture (in a final volume of 20 μl) contained: 2 μl cDNA sample, 10 μl SYBR® Premix Ex Taq™ II (Takara Bio Inc.), and 400 μM of each gene-specific primer. Reactions were set up manually. Without a template, no Cq could be determined since it never passed the threshold line. Serial 5-fold dilutions of cDNAs were used to calculate the standard curve and measure the amplification efficiency for each target and reference gene with the LightCycler® 480 Software version 1.2. The qPCR specificity was confirmed by the sharpness of the peak appeared in dissociation curve analysis. Water was used as non-temperature control, and no significant amplification was observed. *Actin* (accession No. KC140126) was used as a reference gene to standardize the signal intensity, according to the previous article (Funayama et al. 2013). In addition, *actin* did not show a dramatic response to the conditions tested in this work. Results were shown as a mean value of three independent samples, and we confirmed that each result was reproducible.

### *In situ* hybridization for *OsASNase* and *OsGS1;1* transcripts

Traverse sections of flag leaves middle portions and of spikelets from rice plants were first placed in a solution of formalin, acetic acid and alcohol (FAA) (1.85% (v/v) formaldehyde, 5% (v/v) acetic acid, and 63% (v/v) ethanol), as described previously (Ohashi et al. 2015). The fixed sections were embedded in paraffin (Sigma, Saint Louis, MO, USA), and stored at 4 °C. The embedded sections were cut into 10 μm-slices using a microtome (Yamato Kohki Industrial Co., Ltd., PR-50, Saitama, Japan). Preparation of digoxigenin (DIG) labeled RNA probes or *OsASNase1*, *OsASNase2*, and *OsGS1;1,* and *in situ* hybridization analyses were performed as described by Ishiyama et al. (2003, 2004). Pre-hybridization and hybridization solution contained 50% formamide, 300 mM NaCl, 10 mM Tris–HCl (pH 7.4), 1 mM EDTA, 100 μg•ml^−1^ herring sperm DNA, 100 μg•ml^−1^ yeast tRNA, and 0.25% (w/v) SDS. For pre-hybridization, the solution was placed onto each slide containing the sections and it was incubated at 55 °C for 1 h. The solution was then replaced with the same solution containing DIG-labeled RNA probes at the concentration of 5 ng•ml^−1^, and the slides were incubated at 55 °C for 16 h. The washing, detection and microscopic observation steps after hybridization were performed as described previously with slight modification (Sakurai et al. 2001; Ohashi et al. 2015). After hybridization, the slides were washed four times with 4× SSC at 50 °C for 10 min. The excess RNA probes were removed by incubation at 37 °C for 30 min in a solution containing 10 mM Tris–HCl (pH 8.0), 500 mM NaCl, 1 mM EDTA, and 20 μg ml^−1^ RNase A. The slides were then washed with the same solution without RNase A for 15 min at room temperature, and twice with 0.1× SSC for 30 min at 55 °C. The products of the *in situ* hybridization were detected with the system of alkaline phosphatase-conjugated anti-DIG antibody, nitroblue tetrazolium chloride and 5-bromo-4-chloro-3-indolyl-phosphate (Roche Diagnostics). After the staining, root sections were observed by optical microscope (Leica DMRB, Leica Microsystems, Tokyo, Japan) with a CCD camera (Leica DFC 500), visualized and photographed by the image handling software, Leica IM50 Image manager (Leica Microsystems).

### Statistics

All data sets were analyzed with the Microsoft Excel add-in software, Ekuseru-Toukei (Social Survey Research Information Co., Ltd. Tokyo, Japan).

## Conclusions

Amino acid measurements revealed that Asp is highly accumulated in both developing grain and flag leaf although the concentration of Asn is much higher than that of Asp in phloem sap. The localization study indicated the distribution of ASNase2 in vascular tissues. Taken together with a lower AS accumulation in grains, AS provides Asn in source organs, e.g. flag leaves and leaf sheaths, for N translocation, while ASNase2 converts Asn to Asp in sink organs, e.g. developing grains.

## Additional files

Additional file 1: Figure S1. Quantitative real-time PCR detection of transcripts for *OsASNase1* (filled column) and *OsASNase2* (opened column) in roots, leaf blades and leaf sheaths at different leaf positions in rice. Rice plants were grown hydroponically for 18 d in water and each organ was harvested individually; whole roots, 2nd leaf blade, 3rd leaf blade, 3rd leaf sheath, and 4th expanding leaf blade. Quantitative real-time PCR analyses were performed using gene-specific primers for *OsASNase1, OsASNase2* and *actin*, respectively, as described in Table 1. The relative contents of these transcripts were normalized against *actin* transcript. Means of four independent samples and standard error values (*n* = 4) are indicated. Significant differences between *OsASNase1* and *OsASNase2* were identified by Student’s *t*-test, and are marked with asterisks: **P* < 0.05 (PDF 401 kb)
Additional file 2: Figure S2. Quantitative real-time PCR detection of transcripts for *OsAS* in rice flag leaves (A) and developing spikelets (B). The flag leaves were harvested during the ripening period from 14 to 35 days after flowering, respectively. Quantitative real-time PCR was performed using gene-specific primers for *OsAS1, OsAS2* and *actin*, like described in Table 1. The relative contents of these transcripts were normalized against *actin* transcript. Means of three to four independent samples and standard error values (*n* = 3 to 4) are indicated. (PDF 405 kb)
Additional file 3: Figure S3.
*In situ* detection of *OsGS1;1* transcripts in rice spikelets at 5 day after flowering. The antisense probe for *OsGS1;1* transcript was hybridized with the sections from spikelets (A). The sense probe for *OsGS1;1* transcript was hybridized with the sections as negative control (B). (PDF 626 kb)

## Abbreviations

AS: Asparagine synthetase
ASNase: Asparaginase
DAF: Days after flowering
EC: Enzyme commission number
GOGAT: Glutamate synthase
GS: Glutamine synthetase
NADH: Nicotine amide adenine dinucleotide
PCR: Polymerase chain reaction
RAP-DB: The rice annotation project database
RT: Reverse transcription
UPLC: Ultra performance liquid chromatography
