# Supplementary figures and images for: A temporal and spatial contribution of asparaginase to asparagine catabolism during development of rice grains
Source: Rice (N Y). 2017 Jan 25;10:3. doi: 10.1186/s12284-017-0143-8 (PMC5267587; doi:10.1186/s12284-017-0143-8)

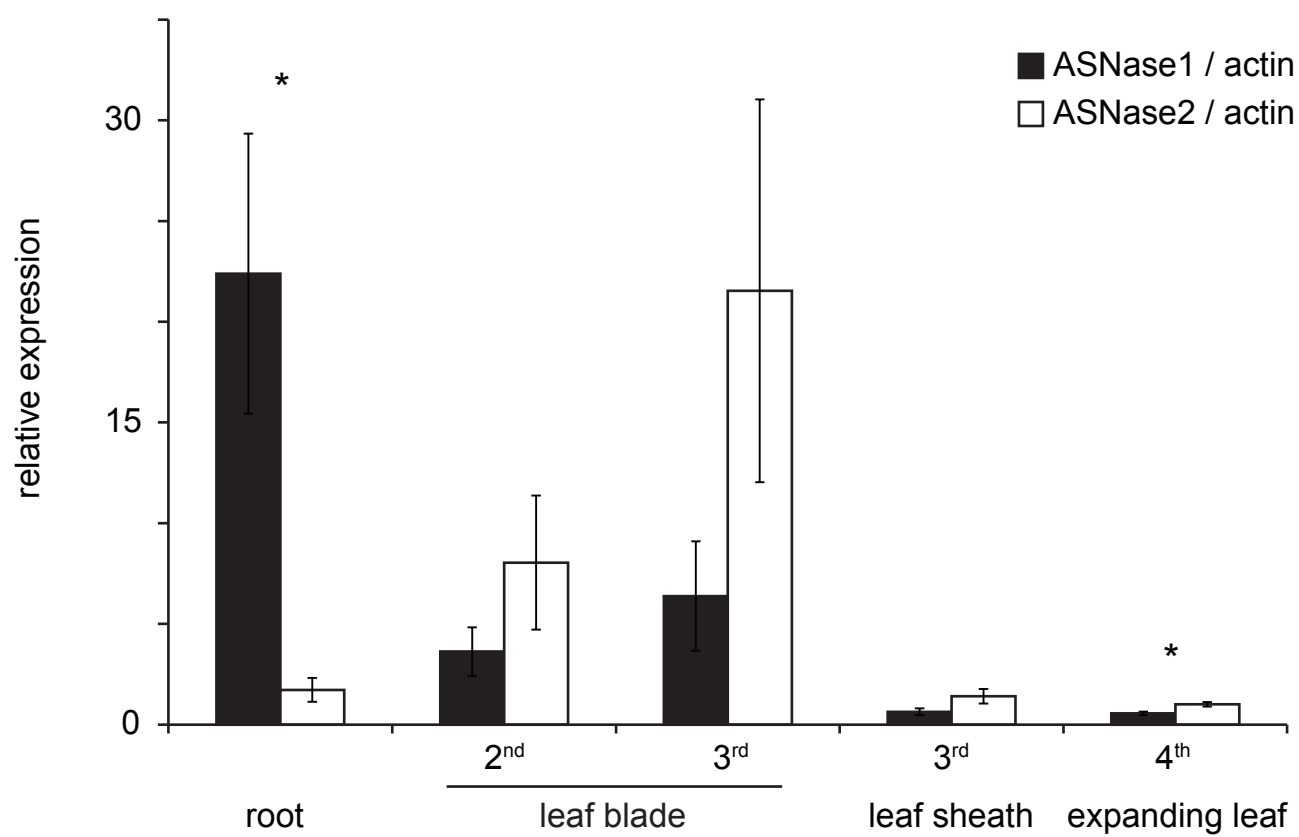

Supplement: Additional file 1: Figure S1. — Quantitative real-time PCR detection of transcripts for OsASNase1 (filled column) and OsASNase2 (opened column) in roots, leaf blades and leaf sheaths at different leaf positions in rice. Rice plants were grown hydroponically for 18 d in water and each organ was harvested individually; whole roots, 2nd leaf blade, 3rd leaf blade, 3rd leaf sheath, and 4th expanding leaf blade. Quantitative real-time PCR analyses were performed using gene-specific primers for OsASNase1, OsASNase2 and actin, respectively, as described in Table 1. The relative contents of these transcripts were normalized against actin transcript. Means of four independent samples and standard error values (n = 4) are indicated. Significant differences between OsASNase1 and OsASNase2 were identified by Student’s t-test, and are marked with asterisks: *P < 0.05 (PDF 401 kb) [file 12284_2017_143_MOESM1_ESM.pdf]

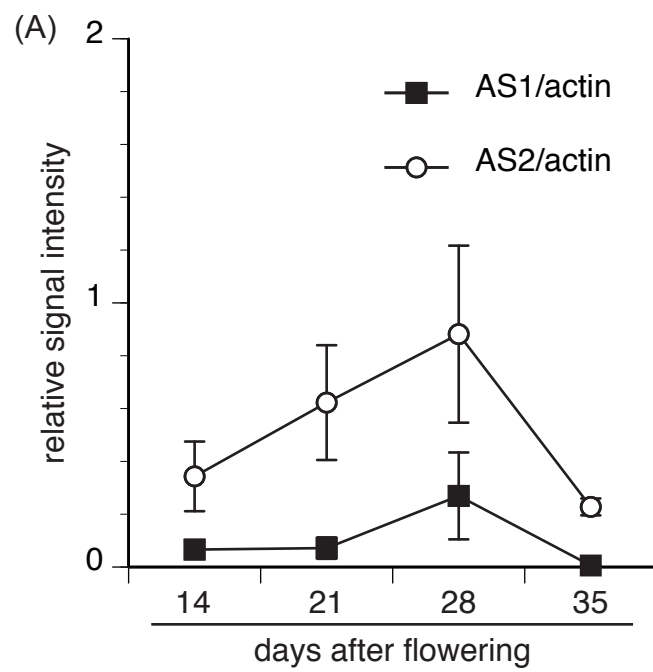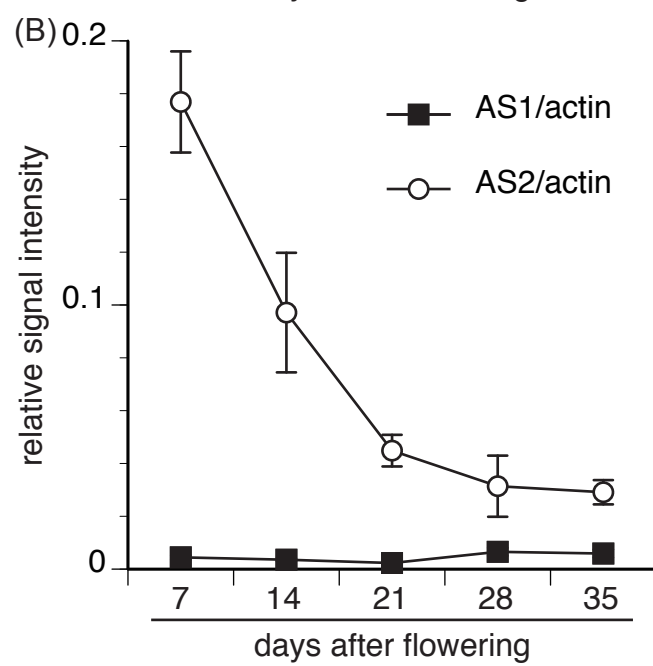

Supplement: Additional file 2: Figure S2. — Quantitative real-time PCR detection of transcripts for OsAS in rice flag leaves (A) and developing spikelets (B). The flag leaves were harvested during the ripening period from 14 to 35 days after flowering, respectively. Quantitative real-time PCR was performed using gene-specific primers for OsAS1, OsAS2 and actin, like described in Table 1. The relative contents of these transcripts were normalized against actin transcript. Means of three to four independent samples and standard error values (n = 3 to 4) are indicated. (PDF 405 kb) [file 12284_2017_143_MOESM2_ESM.pdf]

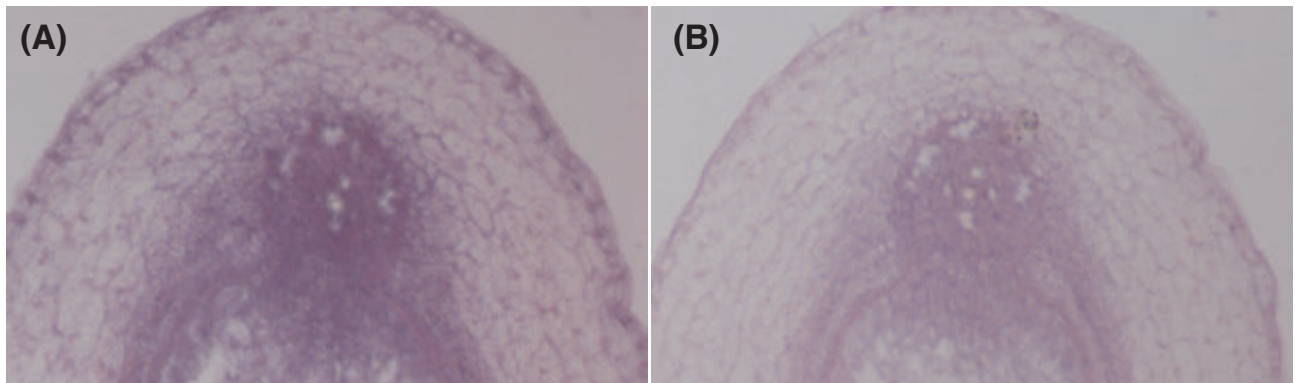

Supplement: Additional file 3: Figure S3. — In situ detection of OsGS1;1 transcripts in rice spikelets at 5 day after flowering. The antisense probe for OsGS1;1 transcript was hybridized with the sections from spikelets (A). The sense probe for OsGS1;1 transcript was hybridized with the sections as negative control (B). (PDF 626 kb) [file 12284_2017_143_MOESM3_ESM.pdf]
